# Intradermal Tests With Drugs: An Approach to Standardization

**DOI:** 10.3389/fmed.2020.00156

**Published:** 2020-05-15

**Authors:** Annick Barbaud, Marie Weinborn, Lene Heise Garvey, Sergio Testi, Violeta Kvedariene, Sevim Bavbek, Holger Mosbech, Eva Gomes, Werner Aberer, Hanneke N. G. Oude Elberink, Maria Jose Torres, Claude Ponvert, C. Ayav, Jimmy Gooi, Knut Brockow

**Affiliations:** ^1^Sorbonne Université, INSERM, Institut Pierre Louis d'Epidemiologie et de Sante Publique, AP-HP.Sorbonne Universite, Tenon Hospital, Departement of Dermatology and Allergology, Paris, France; ^2^Dermatology Department, CHU Nancy, Brabois Hospital, Vandœuvre-lès-Nancy, France; ^3^Dermatology Department, Valenciennes Hospital Avenue Desandrouin, Valenciennes, France; ^4^Allergy Clinic, Department of Dermatology and Allergy, Copenhagen University Hospital, Copenhagen, Denmark; ^5^Immuno-Allergological Department, Firenze Hospital, Firenze, Italy; ^6^Clinic of Infectious, Chest Disease, Dermatovenerology and Allergology, Vilnius University, Vilnius, Lithuania; ^7^Division of Immunology and Allergy, Department of Pulmonary Disease, Ankara University, School of Medicine, Ankara, Turkey; ^8^Serviço de Imunoalergologia, Centro Hospitalar Do Porto, Porto, Portugal; ^9^Department of Dermatology, Medical University of Graz, Graz, Austria; ^10^Department of Allergology, University Medical Center Groningen, University of Groningen, and Groningen Research Institute for Asthma and COPD, Groningen, Netherlands; ^11^Allergy Service, Málaga Regional University Hospital-IBIMA-ARADyAL, Málaga, Spain; ^12^Department of Paediatrics, Pulmonology & Allergy, Paris Descartes University, Necker-Enfants Malades Hospital, Paris, France; ^13^University Hospital of Nancy, Clinical Investigation Center - Clinical Epidemiology, Nancy, France; ^14^Department of Clinical Immunology and Allergy, King's College Hospital, London, United Kingdom; ^15^Department of Dermatology and Allergy Biederstein, Technische Universität München, Munich, Germany

**Keywords:** drug allergy, intradermal test, amoxicillin, standardization, specificity of drug skin tests

## Abstract

**Background:** Intradermal tests (IDTs) are performed and interpreted differently in drug allergy centers making valid comparison of results difficult.

**Objective:** To reduce method-related and intercenter variability of IDTs by the introduction of a standardized method.

**Materials and methods:** In 11 centers of the European Network for Drug Allergy, IDTs were prospectively performed with saline and with amoxicillin (20 mg/ml) using (1) the local method and (2) the standardized European Network in Drug Allergy (ENDA) method (0.02 ml). The diameters of the initial injection wheal (Wi) for the different volumes and sites injected obtained from each center were analyzed.

**Results:** The most reproducible method was to fill a syringe with test solution, then expel the excess fluid to obtain exactly 0.02 ml. The median Wi diameter with 0.02 ml injection using the standardized method was 5 mm [range 2–10 mm; interquartile range (IQR) 5–5 mm; *n* = 1,096] for saline and 5 mm (range 2–9 mm; IQR = 4.5–5 mm; *n* = 240) for amoxicillin. IDT injection sites did not affect the Wi diameter. Training improved precision and reduced the variability of Wi diameters.

**Conclusion:** Using the standardized IDT method described in this multicenter study helped to reduce variability, enabling more reliable comparison of results between individuals and centers.

## Introduction

Allergy skin testing is essential for the correct diagnosis of immediate and delayed drug hypersensitivity (DH). It is also used to identify alternative drugs for patients with positive skin or provocation tests with suspected drugs. The intradermal test (IDT) is the most sensitive skin test and may be used when soluble forms of the drugs are available. A questionnaire survey in 2004 (1) within the European Network in Drug Allergy (ENDA), the Drug Allergy Interest Group of the European Academy of Allergy and Clinical Immunology (EAACI), showed differences in performing drug allergy investigations. Guidelines such as those by the European Society of Contact Dermatitis (ESCD) (2), the EAACI (3), anesthesiology societies (4, 5), and the United States of America (6) differ in their recommendations (Table 1), making valid comparison of the results between centers virtually impossible. A position paper providing guidelines on drug concentrations for skin testing was published in 2013 (7), but at the present time, there is no consensus on the methodology and interpretation of drug IDT. The drug concentration, the method used, and the criteria for positive skin tests all influence the sensitivity and specificity of IDT; consequently, thresholds for specific results may vary between different centers (8, 9). Barbaud et al. (8), using the ESCD guideline, showed that the highest specific concentration before causing an irritant reaction for cefotiam was 10 mg/ml and, for cefotaxime, 25 mg/ml, but Torres et al. (9) in the EAACI Interest Group on DH position paper recommended using 1–2 mg/ml for cephalosporin IDT.

**Table 1:** Comparison of international guidelines published for performing drug intradermal tests.

	**ESCD (2)**	**EAACI (3)**	**BSACI (4)**	**SFAR (5)**	**Macy et al. (6)**
Volume injected	0.04 ml sequential dilutions in saline or phenolated saline	0.02–0.05 ml	0.03 ml	0.02–0.05 ml	0.02 ml with a 27-gauge tuberculin
Measurement of the Wi	Yes Raising a wheal of 4–6 mm	Yes Raising a bleb of 3 mm	Yes Raising a bleb of 4–6 mm	Yes Wheal ≤ 4 mm	No
Time interval to immediate skin test reading (minutes)	30	15–20	20–30	20	15
Criteria for immediate positivity	Wheal ≥ 10 mm	W20 ≥ Wi+ 3 mm with surrounding flare	A wheal that is ≥3 mm larger than the initial bleb with surrounding flare	W20 ≥ Wi × 2	Wheal ≥5 mm with a surrounding erythema
Criteria for delayed positivity	Papule at 24 h	24–72 h infiltrated erythema	Not defined	Not defined	Not defined
Site	Extensor surface of the arm	Volar aspect of the forearm (or other regions)	Not defined	Back, arm or forearm	Not specified
Negative control with saline	Yes	Yes	Yes	Yes	Yes, with Tris-buffered saline

*ESCD, European Society of Contact Dermatitis; EAACI, European Academy of Allergy and Clinical Immunology; BSACI, British Society for Allergy and Clinical Immunology, Societe Francaise d'Anesthesie et Reanimation*.

*Wi, diameter of initial wheal just after injection; W20, diameter of the wheal 20-min post-injection*.

A questionnaire survey on the skin test methods used by different centers in the ENDA group was carried in 2008/2009. It showed a wide variation in the method used and in the interpretation of the results. This led to the setting up of a multicenter study comparing local IDT methods with the proposed new standardized IDT method based on the ENDA consensus and to determine if the standardized method will minimize the intercenter variability in performing IDT. As the initial results and analysis had showed large variation in the diameter of initial wheal just after injection (Wi) readings obtained in the different centers, a practical session was organized during an ENDA meeting in one participating hospital. We noted the different ways of filling the syringe and differences in the injection needle gauge, injection, and test measurement methods. A standardized IDT (the Guideline) was written and validated by all coauthors.

## Materials and Methods

All tests performed in this prospective descriptive study were part of routine investigations in patients who had been referred for investigation of DH, and no additional tests were carried out. Information on the methodology used to carry out the drug IDT was collected from databases of 11 departments in Europe with special interest in DH. The following data were recorded: age and gender of the patients, the method for filling the syringe, the injected volume of saline and amoxicillin (AX), the injection site, and the diameter of Wi and 20 min post-injection. For AX (20 mg/ml), the parenteral AX powder (the manufacturer may vary depending on the center and dispensed by local hospital pharmacy) was dissolved in sterile isotonic saline and used within 2 h of the IDT as recommended (9).

### Questionnaires on the Methods for Doing IDT

A questionnaire was sent by email to all the members of the ENDA group. The main questions aimed at highlighting differences in the local practice between centers, and the returns are summarized in Table 1.

### Evaluating Injection Volume Obtained by Using Different Syringe Sizes and Syringe-Filling Methods

Two methods, used in participating centers, to fill a syringe with 0.02 ml normal saline (NS) were evaluated. In Nancy, three nurses specialized in drug allergy workup drew up exactly 0.02 ml into a 1-ml syringe using a 25-G needle, which was then emptied into a small vial (Method 1). Another nurse filled a syringe with 0.05–0.07 ml saline, then expelled the excess fluid and air bubbles to obtain exactly 0.02 ml, which was similarly emptied into a vial (Method 2). The two procedures were repeated 10 consecutive times. The weight of the NS collected by both methods was determined using a precision scale (KERN EW/EG version 2.4 11/2006) and then converted into milliliters. Method 2 was also carried out in Copenhagen using a 1-ml syringe as in Nancy, and a 0.5-ml syringe and a 27-G needle in both instances.

### Comparing Non-standardized Methods With Injection of a Standardized Volume

The first part of the study was done in order to determine if adhering to previously published international guidelines minimizes intercenter variability in performing drug IDT; each participating center performed IDT using its local IDT protocol with NS and AX (20 mg/ml) test solutions. In the second part of the study, the ENDA method, injecting a fixed volume (0.02 ml) of AX or NS, was carried out on additional new patients. We also analyzed if the injection site, the syringe size, and the needle gauge influenced the size of Wi.

The injection sites used for saline or AX IDT were the lateral aspect of the upper arm (UA) and/or the flexor aspect of the forearm (FA), and, in a limited number of patients, the back (B). All centers used 25- to 27-G needles except one, which used 30-G needles. The injection sites were inspected just after the injection, with the measurement of the Wi and at 20 min post-injection for wheal (W20) and erythema (E20), respectively, and their diameter was measured as recommended (2–5).

### Analysis of Wi When Standardized IDT Is Performed by Individual Tester Injecting 0.02 and 0.03 ml Saline, Respectively

As the preliminary results showed that the test volumes injected varied among allergy centers from 0.02 to 0.05 ml, it was decided to compare the most used volume of 0.03 ml with the proposed lower volume of 0.02 ml NS. This was carried out by trained operators using the standardized IDT method on volunteer subjects in six centers.

The results obtained, the Wi obtained using the local and the standardized IDT methods on different injection sites and volume injected, were subject to chi-square and Kruskal–Wallis tests and non-parametric data by Wilcoxon test. The differences in wheal sizes were considered statistically significant if the *p*-value was ≤0.05. The statistical analysis was performed using SAS software, version 9.2.

## Results

### Questionnaires on the Methods for Doing IDT

All centers answered that they followed the ESCD and/or EAACI guidelines (2, 3), but no two centers carried out and interpreted the IDT in the same way (Table 2). Even if the two European guidelines recommended injecting a given volume (between 0.02 and 0.05 ml) (2, 3), 12/20 centers did not use a fixed volume but injected a volume to produce the targeted Wi diameter.

**Table 2:** Results of Questionnaire Survey of Drug Intradermal Test (IDT) methods used in 20 European allergy centers.

**Questions**	**Answers**
Number of IDT done per year	30–6,000 IDT
Do you use dissolved and filtered drug solution for IDT?	Yes: 5/20 (crushed pills or other non-injectable forms of the drugs, diluted in saline then filtered) No: 15/20
Which solvent do you use?	Saline or phenolated saline: 16/20 Sterile distilled water or the solvent recommended as diluent for the infusion: 4/20
What volume do you inject?	No fixed volume but a volume to raise a wheal: 12/20A fixed volume: 0.02 ml: 1 0.03 ml: 3 0.04 ml: 3 0.05 ml: 1
Site of injection	Upper arm: 4 Forearm: 14 Back: 1 Non-specified: 1
Measurement of the Wi	Yes: 15/20 No: 5/20
What are your criteria for a positive immediate reading?	The existence of a given diameter of W20: 4 centers (3–5 mm depending on the centers) The existence of a given diameter of erythema at 20 min (E20) 3 mm ≥: 6 mm depending on the centers (2 centers also consider the W20). W20 ≥ Wi + 3 mm: 6 centers

*Wi, mean diameter of the wheal (bleb or papule) immediately after ID injection*.

*W20, mean diameter of the wheal or the wheal developed at 20 min*.

*E20, mean diameter of the erythema developed at 20 min*.

In addition to the answers summarized in Table 2, the centers may differ on the syringe size and the gauge of the needle used, on the way syringes are filled with the test solution, and in the training for IDT. Some centers also used crushed pills, diluted in saline then filtered, for performing IDT, which was not recommended by any previous guidelines.

### Evaluating Injection Volume Obtained by Using Different Syringe Size and Syringe-Filling Method

**Figure 1:**
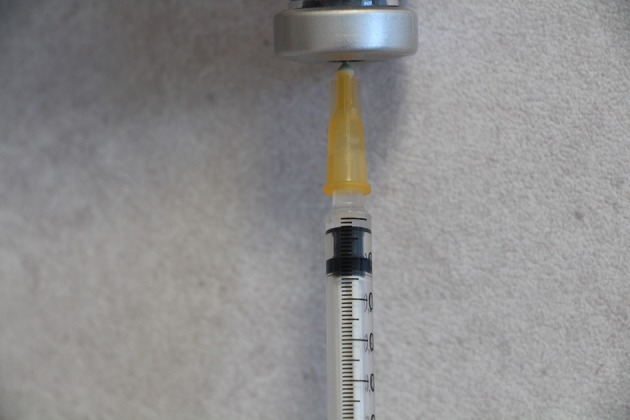
Tuberculin syringe with a 25-G needle and a flat-end plunger drawn up with 0.02-ml solution.

Drawing up a larger volume and expelling excess solution to the required volume gave more reproducible result (mean 0.024 ml, SD = 0.002). This method of syringe filling was adopted into the standardized IDT method (Figure 1). The needle gauge did not appear to affect the injection volume obtained as shown by the results acquired in Copenhagen and Nancy. Copenhagen (27-G needle and 1-ml syringe) mean volume = 0.027 ml (range 0.019–0.037), SD = 0.0.0037. Nancy (25-G needle and 1-ml syringe) mean volume = 0.027 ml (range 0.012–0.037), SD = 0.0035. When a 0.5-ml syringe instead of a 1-ml syringe was used to draw up the solution, the mean volume was less and the standard deviation lower, 0.023 ml (range 0.017–0.027), SD = 0.0019.

### Comparing Non-standardized Methods With Injection of a Standardized Volume

Seven centers performed local non-standardized NS IDT with injection volumes that ranged from 0.02 to 0.05 ml and also with a fixed volume of 0.03 ml (Table 3). Local center IDT methods of skin testing were based on previously published guidelines (2, 3).

**Table 3:** Median injection wheal (Wi) diameter for intradermal tests with saline using non-standardized individual protocols, demonstrating large inter-center variations.

**Center**	**Porto**	**Vilnius**	**Ankara**	**Paris (Necker)**	**Firenze**	**Nancy**	**Munich**	**Total**
Mean age (yrs.)	8	47	43	9	45	55	32	42
**Site**								
UA (*n*)			15	9		24	24	72
FA (*n*)	6	32	11		99	22	24	194
B (*n*)			28				24	52
**Injected volume**								
0.02 ml (*n*)			3				36	39
0.03 ml (*n*)	6	22	48	9	99	46	36	266
0.04–0.05 ml (*n*)		10	3					13
Total (*n*)	6	32	54	9	99	46	72	318
Median Wi diameter (R) mm by center	6 (5–8)	5 (3–11)	3 (2–4)	8 (7–9)	5 (4–6)	5 (3–7)	4.5 (3–7)	Wi diameter Median 5 mm Range (2–11) IQR 4–5 ^**^p < 0.0001

*UA, Upper Arm; FA, Forearm; B, Back; n, number of tests; R, range minimal and maximal; IQR, interquartile range*.

** *The variables were subject to a chi-squared test for the qualitative variables, a Kruskal-Wallis test for the quantitative variables*.

There were large intercenter and interindividual variations in the Wi when local IDT methods were compared. This is likely to be due to the different ways the injection volume was measured and the injection technique. There was a statistically significant difference in the mean Wi diameter across centers even when a fixed volume of 0.03-ml injection was used for IDT (*p* < 0.0001). The overall median Wi was 5 mm (*n* = 318) with a range of 2–11 mm, variation in mean Wi of 3–8 mm, and interquartile range of 4–5 mm.

### Investigation of Whether Standardized IDT Method Reduces Intercenter Variability

During an ENDA meeting, syringe filling, differences in the injection needle gauge, injection technique, and reading of the skin reaction were evaluated.

Needles of 25–30 G did not affect the Wi produced. However, the volume of the syringe used affected the actual volume drawn up into the syringe, probably due to the dead volume of the syringe. In addition, a 0.5-ml syringe has wider spacing between markings, which enables a more accurate measurement of volume of the test solution. Unfortunately, the 0.5-ml syringe is not available throughout Europe. We also consider the variability due to syringe size to be limited. For the standardized method, we, therefore, advised that either 0.5- or 1-ml syringes can be used for IDT, subject to local availability.

To help decide on the test volume to be used in the study, NS IDT was performed on the skin of the coauthors of this study. It was noted that larger injection volumes (>0.02 ml) tended to produce very big wheals. A standardized IDT method with a description of each step, using 0.02-ml injection, was proposed and agreed by the study group (Table 4).

**Table 4:** Summary of proposed ENDA protocol for performing and reading drug intradermal test.

1. IDT must be performed, following negative prick tests, using pharmaceutical grade human drugs in injectable form. IDT is contraindicated in severe cutaneous adverse drug reactions
2. Syringe size and needle gauge: • Tuberculin syringe of preferably 0.5 ml or if not available, 1-ml volume • Needle gauge of 25, 27, or 30 G • Same fixed or new needle can be used for test IDT
3. Injection technique: - Adopt sterile techniques. - Fill syringe with test solution; change the needle if not fixed. Tap the barrel of the syringe to make the air bubble rise to the needle end of the syringe. Expel air bubble and excess volume pushing the plunger to the 0.02-ml mark on the barrel (Figure 1). A syringe with a flat end plunger is better than one with a tapered end to help measure the volume of the test solution drawn into the syringe. - With the bevel of the needle facing upward, pierce skin tangentially in the upper dermis (at about 10° angle to the skin surface). - Then slowly inject the measured volume intradermally.
4. Control: - After intradermal injection of 0.02 ml of saline or test solution at recommended concentration, an injection wheal measuring 4.5- to 5.5-mm diameter should form. - If no clear wheal forms, repeat injection.
5. Record all injected solutions, batch number, and map of injection sites. Draw around and/or measure the diameter of the immediate injection wheal (Wi). If you surround the wheal with ink, always measure the inner diameter.
6. Read the IDT after 20 min. Measure wheal (W_20_) and surrounding erythema (E_20_).
7. If the wheal is not round, measure the length (L), then the width (w) taken perpendicularly, in the middle of the axis length Wi = (L+w)/2.
8. In patient records and publications, IDT results must be recorded as follows: Wi, W_20_, and E_20_.
9. At 20 min, the IDT is considered positive only if there is a wheal, W_20_ ≥ Wi + 3 mm and surrounding erythema, E_20_.
10. For delayed reactions read at 24, 48 h, or later (please specify the time interval). IDTs are considered positive when there is an erythematous induration or swelling at the injection site.

Eight centers performed IDT using 0.02-ml saline, and six of these, in addition, performed IDT with 0.02-ml AX (20 mg/ml).

IDT performed with standardized method syringe filling (Figure 1) and 0.02-ml saline on 1,096 patients (Table 5) showed that a significant difference in the Wi persisted between centers (*p* < 0.0001). However, differences in the mean and median diameters between centers were reduced, with variation in the mean Wi (4.5–5.4 mm) in the range 2–10 mm, with a median diameter of 5 mm (IQR 5–5 mm).

**Table 5:** Diameter of IDT injection wheal (Wi) after injecting 0.02 ml saline and amoxicillin (20 mg/ml) using standardized method for drug intradermal tests.

	**Porto**	**Vilnius**	**Ankara**	**Firenze**	**Nancy**	**Malaga**	**Graz**	**Groningen**	**Total**
Saline number tested	26	87	78	118	734	6	24	23	1,096
Age	10	51	48	46	59	28	28	44	43
**Site**									
UA	0	0	0	0	409	0	8	0	417
FA	26	87	78	118	133	6	8	23	479
Back					192		8		200
Median Wi diameter (R) mm	5 (4–6)	5 (3–10)	5 (4–6)	5 (3–6)	5 (2–7)	5 (4–6)	5.4 (4–7)	4.5 (3–6)	Wi diameter Median 5 mm Range (2–10) Q1 = 5, Q3 = 5, IQR = 0, ^**^p < 0.0001
Amoxicillin number tested	3	23	0	86	88	17	0	23	240
Age	5	51		45	53	35		44	48
**Site**									
UA	0	0	0	0	88	0	0	0	88
FA	3	23	0	86	0	17	0	23	152
Median Wi diameter (R) mm by center	5 (5–6)	6 (4–9)		5 (3–6)	5 (2–7)	6.4 (4–7)		5 (4–7)	Wi diameter Median 5 mm Range (2–9) Q1 = 4.5, Q3 = 5, IQR = 0.5, ^**^p < 0.0001

*R, range minimal and maximal; IQR, interquanrile range; Wi, injection wheal; UA, Upper Arm; FA, Forearm; B, Back*.

** *The variables were subject to a chi-squared test for the qualitative variables and a Kruskal-Wallis test for the quantitative variables*.

IDT with standardized syringe filling with 0.02-ml AX performed on 240 patients gave a mean Wi diameter of 5.2 mm and a median Wi of 5 mm (range 2–9 mm, IQR 4.5–5 mm). The difference in Wi between centers remained statistically significant (*p* < 0.0001) (Table 5).

There was no correlation of wheal size with age or sex with either local methods or standardized IDT.

### Analysis of Wi When Standardized IDT Is Performed by Individual Tester Injecting 0.02- and 0.03-ml Saline, Respectively

The Wi obtained by an individual tester injecting 0.02- and 0.03-ml NS, respectively, using the standardized IDT was compared in six different centers (Table 6). With 0.02-ml NS injection volume, the Wi mean diameter was 5.1 mm (range 3–8 mm) and median = 5 mm (IQR 4.5–5 mm). When 0.03-ml NS was injected, the mean Wi was 6.2 mm (range 3–8 mm) and median = 6 mm (IQR 5.5–6 mm) (Table 6).

**Table 6:** Comparison of diameter of IDT injection wheal (Wi) after injection of saline (0.02 ml or 0.03 ml) by trained operators using the standardized method and syringe size used.

	**Groningen**	**Firenze**	**Porto**	**Nancy**	**Malaga**	**Munich**	**Total**
Number of volunteers	23	11	10	15	10	8	77
Mean age	44	44	34	30	48	30	30
**Site (*****p*** **=** **0.4735)**							
UA (*N*)	0	0	0	30	0	0	30
FA (*N*)	46	22	20	0	20	16	124
**Wi diameter (mm) with injection volume (ml)**							
0.02 ml	5 (3–6)	5 (4–7)	5 (4–6)	5 (5–6)	6 (5–7)	5 (5–8)	5 (3–8)
0.03 ml	6 (3–6)	6 (5–8)	6 (6–7)	6 (6–6)	7 (6–8)	7 (6–7)	6 (3–8)
**Syringe size (ml)**							
0.5 ml (*n*)	46	0	20	0	0	0	66
1 ml (*n*)	0	22	0	30	20	16	88

*N, number of test; M, median; R, range; SD, standard deviation; UA, Upper arm; FA, Forearm; IQR, interquartile range; No difference of Wi according to the site of injection (p = 0.4735)*.

Standardization of IDT produced larger Wi. For the 0.03-ml injection volume, the mean Wi was 6 mm (Table 6) compared to 5 mm before standardization (Table 3). However, the smaller injection volume of 0.02 ml produced smaller Wi (5 mm) (Table 6).

### Injection Site Does Not Affect Wi Readings

The Wi diameter was not affected by the injection site when the standardized IDT method was used. NS IDT was performed on two or more injection sites (UA, FA, B) in three centers (Ankara, Nancy, and Munich). There was no significant difference in the Wi diameter obtained in the different injection sites (*p* > 0.05) (Table 3). In addition, the Wi diameter obtained using the standardized IDT with NS and AX at the recommended concentrations (8–10) performed on the UA, FA, and B was not significantly different and suggested that variation of the test results between centers was independent of the injection site and the drug used. Differences in Wi in different sites in the same individual patients were not compared.

## Discussion

IDTs are essential for the diagnosis of DH. This study demonstrates significant differences in the IDT methods used, in the volume injected, and in the Wi diameter obtained by the study centers. It shows that standardization of the IDT procedure and injection volume produced improved, reproducible, and more comparable skin test results. Tester training had a positive influence on precision and reduced variability. The identification of several causes of variability in the performance of IDT enabled an IDT standardized method to be proposed (Table 4). The adoption of the proposed method and training should lead to more reproducible and comparable results between centers and clinical studies.

In spite of the many guidelines published, our questionnaire survey of 20 allergy centers in ENDA showed that the majority did not follow published guideline recommendations. Indeed, most of the centers (12/20) did not inject a fixed volume as recommended by all guidelines (2–6), but injected a volume to achieve the target Wi. Using a fixed volume of an IDT drug solution of known concentration means injecting a known and fixed quantity of the tested drug. Drug IDT could induce immediate or delayed flare reactions in addition to the wheal (11, 12). The incidence of these flares may depend on the method, the concentrations, and the volume injected. Three flares were observed among 30 patients with IDTs for cutaneous adverse drug reactions (11). The incidence of systemic reactions in patients with positive skin tests to penicillin varies from 0.7 to 9.4% (12, 13). Even with a fixed injection volume, the diameter of Wi could be affected by the age of the individual and the degree of skin atrophy. Injecting a fixed test volume would allow more robust comparison of IDT results between individuals and centers. As illustrated in Table 1, the five guidelines vary in the volume of drug injected, the target Wi diameter, the time before reading the immediate skin reaction, the criteria for an immediate and delayed positive test reading, and the site for performing IDT. The differences in the Wi diameter following injection of test volumes used in the guidelines and in our standardized method are stark.

With the ENDA IDT standardized method, the 0.02-ml injection volume produced a mean Wi of 5.1 mm (range 3–8 mm), whereas 0.03 ml produces a mean Wi diameter of 6.2 mm (3–8 mm), median = 6 mm. With the ESCD guidelines showing that injecting 0.04 ml results in raising a Wi of 4–6 mm (2), the EAACI guidelines show that injecting 0.02–0.05 ml produced a Wi of 3 mm (3), and the SFAR guidelines show that injecting 0.02–0.05 ml caused a Wi ≤ 4 mm (5). Such differences make the comparison of IDTs performed following different guidelines of doubtful value. The BSACI guideline uses a fixed volume of 0.03 ml. The resulting wheal, a median Wi of 6 mm, is similar to that obtained by the ENDA standardized method. However, there is a difference in the time when the immediate test reading is taken, 20–30 min with the BSACI guideline and 20 min with the ENDA standardized method.

Macy et al. (6), in their IDT protocol, used 0.02 ml and took the reading of the immediate reaction 15 min after the injection, which was considered positive when the wheal is ≥5 mm. In a recent paper, the similarities and differences between Europe and North America in the approach to the diagnosis of DH reactions have been highlighted (14). However, the method for doing and reading IDT, which we have shown to be different among ENDA allergy centers, is not in the list of differences between the two continents.

With the standardized IDT method, the 0.02-ml injection volume produced a mean Wi of 5.1 mm, whereas 0.03 ml and above produced a Wi diameter of 6.2 mm (3–8 mm). Due to the small risk of sensitization and anaphylaxis induced by IDT (9), it is considered good practice to inject a small as possible volume of potential drug allergen that will produce a test wheal that enables accurate reading of the diameter. That is why we have recommended that 0.02 ml of non-irritating test allergen solution should be used for IDT.

Variability could also be caused by differences in measuring wheal sizes and differences in the depth of injection. The somewhat surprising but interesting finding that the injection site did not significantly affect Wi needs further evaluation. It appears that IDT is a more complicated and variable method than previously acknowledged and that detailed recommendations and training are needed for method consistency and reproducible results and interpretation. Intertester variability could be reduced by having designated trained members of staff to perform IDT (15).

We hope that these highly detailed ENDA guideline for performing IDT will help to standardize the IDT method. We envisage that further studies will be necessary using this standardized method to determine if Wi could be affected by the age of the patient, the test site, and skin atrophy. It would also be interesting to determine the negative predictive value of IDT in using different criteria for their positivity W20 ≥ Wi+ 3 mm, W20 ≥ Wi × 2 or W20≥ a fixed diameter of 10 or 5 mm.

## Data Availability Statement

All datasets generated for this study are included in the article/supplementary material.

## Ethics Statement

Ethical review and approval was not required for the study on human participants in accordance with the local legislation and institutional requirements. The patients/participants provided their written informed consent to participate in this study.

## Author Contributions

AB, MW, ST, VK, SB, LG, HM, EG, WA, HE, MT, CP, JG, and KB: collecting, data, and corrections. AB, MW, and JG: writing the document. CA: statistics.

## Conflict of Interest

The authors declare that the research was conducted in the absence of any commercial or financial relationships that could be construed as a potential conflict of interest.
